# Stress-resistant corals may not acclimatize to ocean warming but maintain heat tolerance under cooler temperatures

**DOI:** 10.1038/s41467-019-12065-0

**Published:** 2019-09-17

**Authors:** Verena Schoepf, Steven A. Carrion, Svenja M. Pfeifer, Melissa Naugle, Laurence Dugal, Jennifer Bruyn, Malcolm T. McCulloch

**Affiliations:** 1 Oceans Graduate School and UWA Oceans Institute, The University of Western Australia, 35 Stirling Highway, Perth, WA 6009 Australia; 2 ARC Centre of Excellence for Coral Reef Studies, The University of Western Australia, 35 Stirling Highway, Perth, WA 6009 Australia; 3 School of Geosciences, University of Edinburgh, James Hutton Road, Edinburgh, EH9 3FE UK; 4 Department of Biology, Heinrich-Heine-Universität Düsseldorf, Universitätsstrasse 1, 40225 Düsseldorf, Germany

**Keywords:** Ecology, Environmental sciences

## Abstract

Naturally heat-resistant coral populations hold significant potential for facilitating coral reef survival under rapid climate change. However, it remains poorly understood whether they can acclimatize to ocean warming when superimposed on their already thermally-extreme habitats. Furthermore, it is unknown whether they can maintain their heat tolerance upon larval dispersal or translocation to cooler reefs. We test this in a long-term mesocosm experiment using stress-resistant corals from thermally-extreme reefs in NW Australia. We show that these corals have a remarkable ability to maintain their heat tolerance and health despite acclimation to 3–6 °C cooler, more stable temperatures over 9 months. However, they are unable to increase their bleaching thresholds after 6-months acclimation to + 1 °C warming. This apparent rigidity in the thermal thresholds of even stress-resistant corals highlights the increasing vulnerability of corals to ocean warming, but provides a rationale for human-assisted migration to restore cooler, degraded reefs with corals from thermally-extreme reefs.

## Abstract (web summary)

Coral populations from thermally extreme conditions may help restore reefs degraded by bleaching. Here, the authors show that these corals can maintain their heat tolerance despite acclimation to colder temperatures but have a limited capacity to acclimatize to ocean warming.

## Introduction

Coral reefs are biodiversity hotspots that provide goods and services to millions of people worldwide, but recurrent mass bleaching events associated with marine heatwaves and climate change significantly threaten the persistence of coral reefs, contributing to their worldwide decline^1,2^. One of the key questions is therefore whether reef-building corals have the capacity to acclimatize and/or adapt to ocean warming, and whether they are able to do so at a rate fast enough to keep pace with intensifying climate change^3–7^. While corals have been able to adapt to changing thermal regimes on millennial timescales such as during glacial to interglacial transitions, marine heatwaves are now occurring on sub-decadal timescales orders of magnitude faster than geologic timescales^8^. Additionally, although corals exist across large latitudinal and seasonal gradients in temperature^9^, their thermal tolerance or bleaching thresholds are typically only 1–2 °C above their local maximum summer temperatures^10,11^. This highlights their extreme sensitivity to rising ocean temperatures and increasingly frequent marine heatwaves^2,8^.

Nevertheless, coral populations with higher heat resistance have recently been discovered in reef environments with naturally extreme, highly variable temperature regimes, such as tide pools in back reef environments^3^ and extreme macrotidal reefs^12^. These naturally heat-resistant corals are ideal model organisms to understand the mechanisms and timescales required to achieve resistance to stressfully high temperatures^3,6,12–14^. Furthermore, they are of critical importance for the persistence of coral reefs because they can potentially provide heat-tolerant alleles through the dispersal of larvae to more thermally sensitive regions, a form of “genetic rescue”^5,15,16^. Finally, they may hold significant potential for pro-active management approaches, such as assisted translocation of heat-adapted genotypes, to restore reefs and improve reef resilience^17,18^. However, critical knowledge gaps exist regarding the ability of these heat-resistant corals to maintain heat tolerance and health outside their native temperature range (e.g. on cooler, thermally stable reefs) and to acclimatize to ocean warming by increasing their thermal tolerance thresholds.

While models predict that genetic rescue via larval dispersal or assisted gene flow could result in improved heat tolerance of cool-adapted populations^5,16^, it remains poorly understood whether heat-adapted migrants from hot, thermally variable reefs will be able to maintain their health on cooler, thermally stable reefs. This is because adaptation to the migrant’s hot and thermally variable native habitat can incur fitness trade-offs (reciprocal home site advantage) in the non-native habitat^19^. Fitness trade-offs associated with local adaptation appear to be widespread in corals and can limit their potential for acclimatization^11,20–22^. Furthermore, experimental evidence for the capacity of naturally heat-resistant corals to maintain their heat tolerance ex situ (e.g. after migrating naturally or via human-assisted translocation to cooler, more thermally stable reefs) is largely non-existent. While coral heat tolerance can have a strong genetic and, thus heritable component^15^, acclimatization also plays a significant role^3^. Lack of highly variable temperatures in the new habitat may therefore lower the heat tolerance of migrants because exposure to higher temperature variability in their native environment enhances resistance to elevated mean temperatures^23,24^.

Finally, rapid acclimatization through phenotypic plasticity could buy time for populations to adapt to accelerating climate change via genetic change^25^. However, it remains largely unknown whether naturally heat-adapted coral populations can acclimatize to progressive ocean warming and improve their superior heat tolerance under climate change. Even heat-resistant coral populations from hot and/or thermally variable reefs have suffered from coral bleaching in recent years^26–28^, highlighting the need to understand whether adaptation to stressfully high temperatures may constrain coral acclimatization capacity. In particular, understanding if these heat-resistant corals have the ability to acclimatize to longer-term ocean warming superimposed upon the extreme temperature variability already encountered in their native habitats will be vital for determining whether they can continue to survive in their native habitats and potentially provide genetic rescue under future climate change.

**Fig. 1:**
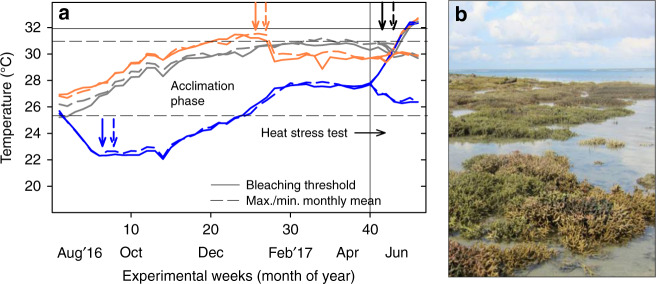
Temperature regimes during the long-term acclimation phase and subsequent heat stress test. **a** Weekly average temperatures of corals maintained at three different temperature regimes and exposed to either constant daily temperature (solid coloured lines) or 4 °C daily temperature variability (dashed coloured lines). For the heat stress test, all treatments were split into ambient control and heated tanks. Coloured arrows indicate when paling/bleaching was observed for the respective treatments. Black arrows represent bleaching in all treatments. **b** Naturally stress-resistant coral communities from the macrotidal Kimberley region exposed during spring low tide. Source data are provided in the Source Data and Supplementary Data 1 files

Here we focus on corals from the extreme macrotidal (10–12 m) Kimberley region in NW Australia, which can withstand frequent, tidal-induced aerial exposure (Fig. 1), daily temperature fluctuations of up to 7 °C and short-term temperature maxima of 37 °C during spring low tides^12,29^. As a result, they have a higher heat tolerance than corals from more moderate thermal environments^12^. Using a long-term (~9 months) mesocosm experiment, we ask whether these corals can acclimate to ocean warming predicted to occur by mid-century (+1 °C) when superimposed on their already thermally extreme native habitat over seasonal timescales. In parallel, we test their potential for natural or human-assisted migration by simulating translocation from their hot, thermally variable reef in the Kimberley region to the much cooler, thermally stable Ningaloo Reef, a reef 1200 km southwest of the Kimberley region. To do so, we expose them to 4 °C cooler temperatures for 9 months to assess whether they could survive and maintain their high heat tolerance on cooler, thermally stable reefs. Finally, we explore the role of temperature variability in promoting acclimatization to warmer/cooler temperature regimes, as fluctuating temperatures often enhance coral heat tolerance^12,23,24^. We show that these corals from thermally extreme reefs are able to maintain their health and native heat tolerance despite long-term exposure to 4 °C cooler temperatures. Although not immune to bleaching^12,26^, this ability, combined with their high tolerance to a range of environmental stressors, makes them promising candidates for human-assisted migration to restore cooler, degraded reefs with corals from thermally extreme reefs. However, these corals have a limited capacity to acclimatize to future ocean warming, painting a more pessimistic picture for the future health of low-latitude corals living close to their upper thermal limits.

## Results

### Simulated translocation to cool, thermally stable reefs

**Fig. 2:**
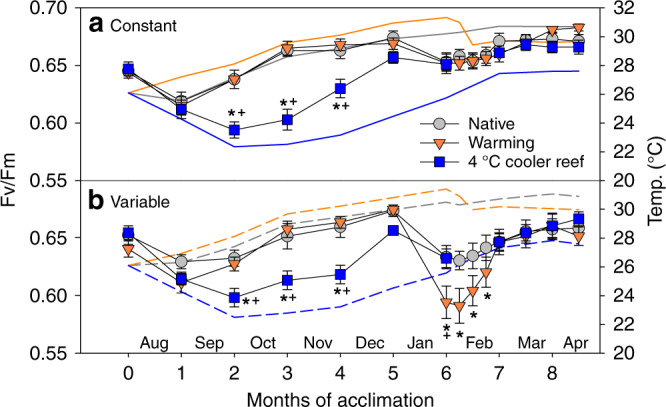
Photochemical efficiency (Fv/Fm) during the acclimation phase. *Acropora aspera* corals were maintained under **a** constant daily temperatures or **b** 4 °C daily temperature variability. Solid and dashed lines represent monthly tank temperatures for each treatment (Table S2). Mean ± 1 S.E.M. is shown. Asterisks indicate a significant difference from the native (control) treatment, whereas plus (+) indicates a significant difference between the warming and 4 °C cooler reef treatments (*p* < 0.05; Tukey post hoc tests). Note that 4 °C cooler reef corals were not measured weekly between months 6 and 7. Source data are provided as a Source Data file

**Fig. 3:**
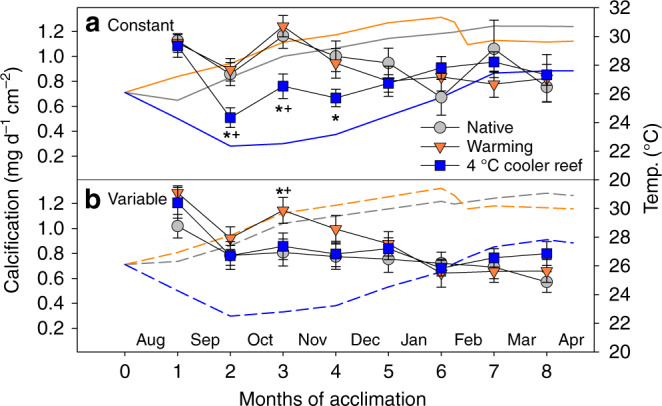
Calcification rate during the acclimation phase. *Acropora aspera* corals were maintained under **a** constant daily temperatures or **b** 4 °C daily temperature variability. Solid and dashed lines represent monthly tank temperatures for each treatment (Table S2). Mean ± 1 S.E.M. is shown. Asterisks indicate a significant difference from the native (control) treatment, whereas plus (+) indicates a significant difference between the warming and 4 °C cooler reef treatments (*p* < 0.05; Tukey post hoc tests). Source data are provided as a Source Data file

**Fig. 4:**
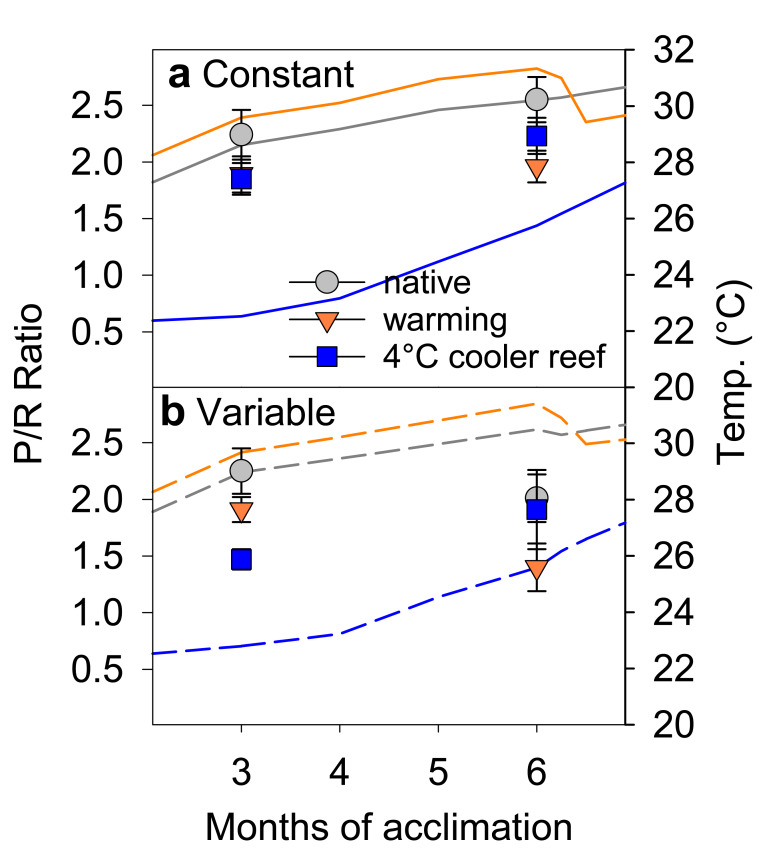
Photosynthesis-to-respiration ratio (*P*/*R*) after 3 and 6 months of acclimation. *Acropora aspera* corals were maintained under **a** constant daily temperatures or **b** 4 °C daily temperature variability. Solid and dashed lines represent monthly tank temperatures for each treatment (Table S2). Mean ± 1 S.E.M. is shown. Source data are provided as a Source Data file

We simulated translocation of naturally stress-resistant *Acropora aspera* corals from a hot, thermally variable reef in the Kimberley region to the much cooler, thermally stable Ningaloo Reef, a reef in NW Australia 1200 km southwest of the corals’ native Kimberley reef (Fig. 1, Supplementary Tables 1 and 2, Source Data 1). Long-term exposure to the much cooler temperatures representative of Ningaloo Reef (~3–6 °C cooler than in their native environment) initially led to significant signs of cold stress during winter (months 2–4). Compared to corals maintained under native temperatures, cold-stressed corals had 19–25% lighter colour indicating significant adjustment of algal symbiont and/or photosynthetic pigment concentrations (Supplementary Fig. 2, Supplementary Table 3). They also had 5–9% lower photochemical efficiency (Fv/Fm; Fig. 2, Supplementary Table 3) and calcified up to 41% less (Fig. 3a, Supplementary Table 3). When cold stress was combined with 4 °C daily temperature variability (Supplementary Fig. 1), this further resulted in 35% lower photosynthesis-to-respiration (*P*/*R*) ratios but this trend was not statistically significant due to data variability (Fig. 4b).

However, cold-stressed corals were nevertheless able to maintain key aspects of coral health and ultimately acclimated rapidly to the cooler temperatures. Even during winter, cold-stressed corals maintained net autotrophy (as indicated by *P*/*R* ratios >1; Fig. 4) and had Fv/Fm and health score values that are typically still associated with healthy corals (Fv/Fm ~0.6, health score ~4.5). Furthermore, acclimation to the cooler temperatures occurred within only 4–5 months as Fv/Fm, health scores and calcification rates were no longer compromised compared to the corals maintained at native temperatures after this time (Figs. 2 and 3a, Supplementary Fig. 2), despite temperatures remaining ~3–5.5 °C cooler.

Daily temperature variability of 4 °C (Supplementary Fig. 1) reduced the negative impacts of cold stress on some aspects of coral health but exacerbated impacts on others. While declines in Fv/Fm and coral health were largely independent of temperature variability (Fig. 2, Supplementary Fig. 2a, b, Supplementary Table 3), only corals exposed to constant cold temperatures experienced significant declines (up to −41%) in calcification (Fig. 3a, Supplementary Table 3). In contrast, *P*/*R* ratios were generally lower under variable compared to constant daily temperatures and also lower in both cold- and warm-acclimated corals compared to the native controls (Fig. 4, Supplementary Table 3).

### Capacity to acclimate to warmer temperatures

We found that corals exposed to 1 °C warmer seasonal temperatures relative to their native environment were able to maintain similar health as the native controls for as long as temperatures stayed within their normal seasonal range (months 1–5). However, as soon as temperatures started to exceed their native maximum monthly mean (MMM) temperatures in spring, heat stress resulted in significant coral bleaching and compromised health. Corals suffered from 17% to 46% colour loss (i.e. bleaching), with greater colour loss occurring under variable compared to constant daily temperatures (−24 to −46% vs. −17%) (Supplementary Fig. 2a, b, Supplementary Table 3). The combination of heat stress and daily temperature variability also resulted in significant declines in Fv/Fm (−3 to −6%), whereas this was not the case under constant daily temperatures (Fig. 2, Supplementary Table 3). *P*/*R* ratios were also compromised in heat-stressed corals (−23 to −30% but the trend was not statistically significant) and were generally lower under variable compared to constant daily temperatures (Fig. 4, Supplementary Table 3). In contrast, calcification rates remained largely unaffected by heat stress (Fig. 3, Supplementary Table 3).

The strong and rapid health decline of corals in the warming treatments in “spring” raised significant concerns regarding their ability to survive on continued exposure to these high temperatures until “summer”, when a heat stress test was planned to assess the impacts of long-term acclimation to warmer/cooler temperatures on coral heat tolerance. *Acropora* corals, including *A. aspera*, are known to rapidly succumb to prolonged heat stress by developing tissue necrosis and sloughing^12^, thus making it highly unlikely that even the corals in the constant hot treatment would have survived continued heat stress for several more months. In order to prevent substantial mortality, temperatures were therefore lowered by 2 °C in the warming treatments at the beginning of February 2017 (Fig. 1a, Supplementary Table 2; see “Methods”). Consequently, heat-stressed corals started to recover over the following weeks, and from month 7–7.5 onwards, their health score and Fv/Fm values were no longer significantly lower than in the corals maintained at native temperatures (Supplementary Fig. 2a, b, Fig. 2).

### Impacts of long-term cold/warm acclimation on coral heat tolerance

**Fig. 5:**
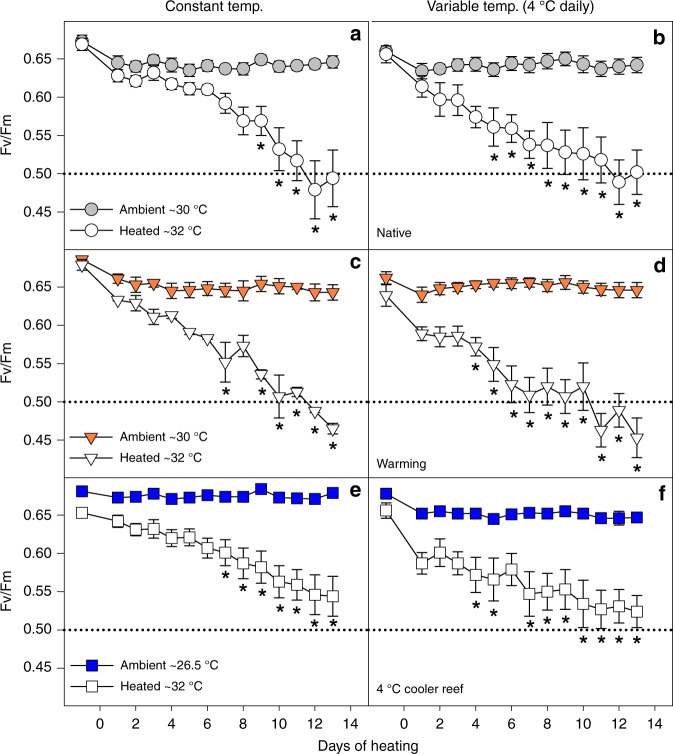
Photochemical efficiency (Fv/Fm) during the heat stress test. *Acropora aspera* corals were maintained under **a**, **c**, **e** constant daily temperatures or **b**, **d**, **f** 4 °C daily temperature variability. The heat stress test followed long-term exposure to three different temperature regimes (native control = circles, warming = triangles, 4 °C cooler reef = squares). Mean ± 1 S.E.M. is shown. Asterisks indicate a significant difference between ambient and heat-stressed corals at each temperature and variability regime (*p* < 0.05; Tukey post hoc tests). The dotted lines were added to highlight differences between treatments. Note that the first time point corresponds to the start of the temperature ramp up (mid-April), approximately 1 month prior to the start of the heat stress test (17 May). Source data are provided as a Source Data file

**Fig. 6:**
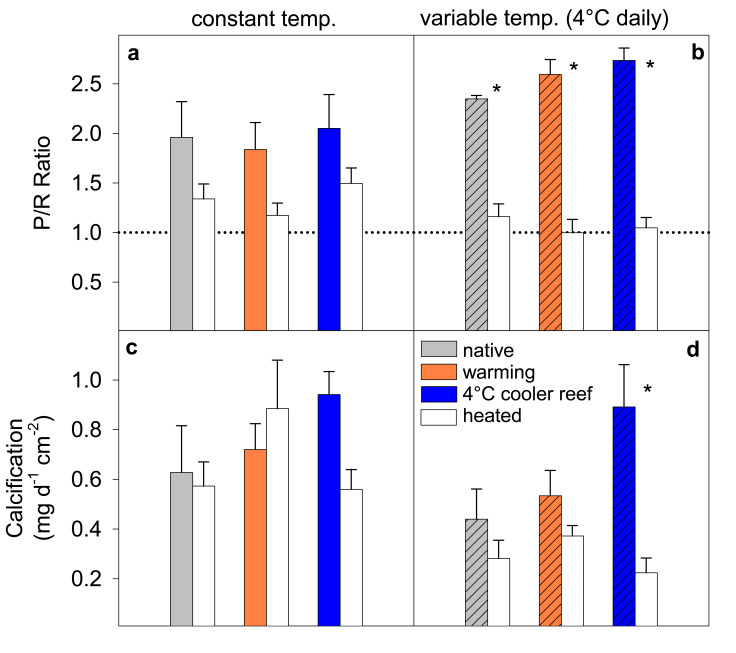
Photosynthesis-to-respiration (*P*/*R*) ratios and calcification rate at the end of the heat stress test. *Acropora aspera* corals were maintained under **a**, **c** constant daily temperatures or **b**, **d** 4 °C daily temperature variability. The heat stress test followed long-term exposure to three different temperature regimes. Mean ± 1 S.E.M. is shown. Grey circles indicate individual data points. Asterisks indicate a significant difference between ambient and heat-stressed corals at each temperature and variability regime (*p* < 0.05; Tukey post hoc tests). The dotted line in **a**, **b** represents the transition from net autotrophy (*P*/*R* > 1) to net heterotrophy (*P*/*R* < 1). Source data are provided as a Source Data file

A 13-day heat stress test was conducted to assess how long-term acclimation to warmer/cooler temperatures in combination with the presence or absence of daily temperature variability affected the heat tolerance of corals from thermally extreme reefs. We used the photochemical efficiency (Fv/Fm) as a highly sensitive indicator of changes in heat tolerance^30^. Exposure to temperatures exceeding the maximum summer temperatures in their native environment by ~1.5 °C (Fig. 1a) led to strong health declines in all heat-stressed corals, independent of daily temperature variability, as shown by significantly lower Fv/Fm values and health scores (Fig. 5, Supplementary Fig. 2c, d, Supplementary Table S4). Heat-stressed corals also tended to have lower *P*/*R* ratios and calcification rates (Fig. 6, Supplementary Table 4) but with more pronounced effects at variable compared to constant daily temperatures (see below). However, long-term acclimation to 1 °C warmer temperatures prior to the heat stress test lowered heat tolerance because heat-stressed warm-acclimated corals experienced greater overall declines in Fv/Fm over the course of the stress test compared to the heat-stressed ambient corals under both constant and variable temperatures (up to −30% vs up to −24%; Supplementary Table 4, Fig. 5a–d).

In contrast, corals acclimated to 4 °C cooler temperatures prior to the heat stress test were able to maintain their native heat tolerance and coped with heat stress similarly to the native corals (under both constant and variable daily temperatures). For example, heat-stressed cool-acclimated corals had higher Fv/Fm than heat-stressed native corals (i.e. >0.5) throughout the stress test and only experienced up to 20% decline in Fv/Fm over the course of the stress test vs up to 26% decline in the native corals (Fig. 5e, f, Supplementary Table 4). This response was in stark contrast to the reduced heat tolerance of the warm-acclimated corals. Colour loss and declines in *P*/*R* of heat-stressed cool-acclimated corals were similar to those experienced by heat-stressed native corals (Supplementary Fig. 2c, d, Fig. 6a, b). In contrast, cool-acclimated corals calcified up to 75% less under heat stress, whereas native corals only calcified up to 36% less (Fig. 6c, d, Supplementary Table 4); however, this was primarily because ambient cool-acclimated corals calcified more than the ambient native corals (Fig. 6c, d, Supplementary Table 4).

Unexpectedly, 4 °C daily temperature variability lowered heat tolerance across all acclimation temperatures (Supplementary Table 4, Figs. 5 and 6, Supplementary Fig. 2c, d). Heat-stressed corals exposed to temperature variability experienced greater overall declines in Fv/Fm over the course of the heat stress test than corals under constant daily temperatures (Fig. 5). Similarly, temperature variability resulted in much lower *P*/*R* ratios (up to −62% vs up to −36% under constant temperatures; Supplementary Table 4, Fig. 6a, b) and lower health scores (up to −69% vs up to −58% under constant temperatures; Table S4, Supplementary Fig. 2c, d). Finally, calcification rates were generally reduced (−36%) in corals under variable compared to constant daily temperatures, independent of acclimation temperature or heat stress (Supplementary Table 4).

We note here that full colony/tank replication was not possible during the heat stress test due to logistical reasons (see “Methods”); thus the effects of heat stress on coral health could potentially also reflect inherent differences in heat tolerance between colonies present in ambient vs heat-stress treatments. However, we consider this unlikely given that (1) these effects, as stated above, were pronounced and statistically significant, and (2) within-treatment variability was much lower than between-treatment variability.

## Discussion

We show here that corals from a thermally variable reef in the extreme macrotidal Kimberley region (NW Australia) had a remarkable ability to maintain their health and high heat tolerance despite exposure to significantly cooler (3–6 °C) temperatures for 9 months. Although they initially experienced cold-stress-induced bleaching in winter, they rapidly acclimated to the cooler temperatures and were able to cope with heat stress as well as the corals maintained under native temperatures. This response differs from another study, where *Acropora millepora* corals transplanted from the warm central to the cool southern Great Barrier Reef also experienced winter bleaching but were unable to recover with significant partial mortality even in surviving corals^11^. This strong negative response was observed despite average temperatures being only 2.2 °C cooler^11^, in contrast to the ~4 °C cooler temperatures in this study. However, the rapid physiological adjustment to cooler temperatures observed here is consistent with other experimental work on *A. yongei*^31^, demonstrating that, at least for some coral species, heat stress appears to be more deleterious than cold stress. These findings have important implications for the ability of more heat-resistant corals to provide “genetic rescue” to coral populations maladapted to rising ocean temperatures, either via natural dispersal of larvae or human-assisted translocation^32^. One important factor to consider, particularly in the context of human-assisted translocation, is that corals transplanted to a cooler reef may have a greater capacity to cope with heat stress as this would occur on the backdrop of their native winter temperatures. Coral bleaching thresholds can be seasonally lower in winter^33^, but we found no evidence for lower bleaching thresholds in response to cold acclimation. Although the occurrence of winter bleaching in our study suggests that the migrant or transplant corals may initially suffer from compromised health, with potential implications for growth and reproduction during the first year^34^, their ability to rapidly adjust their physiology to the cooler temperatures and ultimately maintain health (as shown by high calcification rates and *P*/*R* ratios) and heat tolerance will be critical for corals migrating to cooler locations to succeed in their new environments. These encouraging results pave the way for new experimental studies and field trials that should consider whether fitness trade-offs could potentially occur in response to other environmental variables encountered in the new environment as migrant or transplant corals often show reduced fitness compared to natives, even within their normal temperature range^11,20,22^. Furthermore, carry-over effects (including parental effects), which are currently poorly understood in corals^35,36^, may result in fitness differences between offspring and their parents.

Our study provides long-term experimental evidence that corals from thermally extreme reefs can retain their superior heat tolerance outside their native temperature range—even under much cooler and thermally more stable temperature regimes. While coral adaptation to local temperature regimes has been demonstrated previously^3,11,15,37^, our study (1) tested the effects of long-term cold acclimation on the heat tolerance of naturally stress-resistant corals from thermally extreme reef environments, (2) assessed this under both constant and variable temperatures, and (3) combined long-term cold acclimation with a rigorous heat stress challenge that tested cold-acclimated corals at their native bleaching thresholds. Future research should now address whether these corals are also able to maintain their heat tolerance and health over the timescales required for currently proposed pro-active management approaches such as human-assisted translocation (i.e. years to decades)^17,18,32,38^ and, importantly, also across generations^35,36^. However, our findings highlight that corals from thermally extreme reefs could potentially be promising candidates for these approaches to sustain cool-adapted coral populations under climate change and restore cooler, degraded reefs.

Naturally stress-resistant Kimberley corals had a limited capacity to acclimatize to future ocean warming, at least over the timescales studied here. Despite acclimation to ~1 °C warmer than their native seasonal temperatures for 6 months, these corals nevertheless suffered from spring bleaching as soon as experimental temperatures exceeded the maximum summer temperature threshold characteristic of their native environment. Furthermore, when allowed to recover for 3 months prior to a further heat stress test, they showed no signs of improved heat tolerance compared to corals maintained at native seasonal temperatures; instead, they bleached sooner and more severely. These findings provide further evidence that long-term acclimatization and/or adaptation to local temperature regimes can lead to rigid thermal tolerance thresholds that limit the capacity of corals to acclimatize to warming oceans^11,20,21^.

Our findings contradict evidence for rapid (1–2 weeks) acclimation to higher temperatures from other studies^6,39,40^. One possible reason is that thermal acclimation can modify the metabolic condition of the coral holobiont^41^. Another possibility is that the spring bleaching lowered their heat tolerance during the subsequent heat stress test due to repeat exposure^42^, potentially mediated via low levels of energy reserves that were not fully recovered by the time the heat stress test was conducted^43^, despite the corals being fully recovered in terms of their visual health score and photochemical efficiency. However, even in this scenario, warm acclimation for 6 months was insufficient to prevent spring bleaching in the first place. This suggests that corals may have a greater capacity to rapidly acclimate to short-term elevated temperatures than longer-term chronic warming and/or that the capacity to improve heat tolerance via acclimatization is more limited in corals from thermally extreme compared to thermally more moderate habitats, as shown for marine invertebrates in the rocky intertidal^44,45^. As a consequence, corals from thermally extreme reefs such as the Kimberley region may have evolved their heat tolerance at the expense of acclimatization capacity and could thus be among those corals most susceptible to progressive ocean warming^12,26,45^. It is important to note though that our study tested for acclimation capacity during the adult life stage; thus future work should focus on whether developmental and transgenerational plasticity^7,35,36^ as well as genetic adaptation across generations^15^ could result in increased resistance to warming, particularly in corals from thermally extreme reefs. Similarly, coral-associated micro-organisms, including members of the family Symbiodiniaceae^46^, may influence the capacity of the holobiont to acclimatize or adapt to rapid climate change. While this was beyond the scope of this study, such work will provide important insights into the mechanisms underlying the holobiont phenotypic responses observed here.

Our study further highlights that mechanisms known to improve heat tolerance, such as short-term pre-conditioning to sub-bleaching temperatures^39,40,47^ and variable temperatures^23,24^, may not necessarily promote acclimatization to ocean warming in populations that exist near their present-day thermal limits. Daily temperature variability surprisingly failed to promote acclimation to 1 °C warmer temperatures and instead exacerbated the impacts of heat stress. Given that these corals originated from an extreme macrotidal, intertidal reef environment characterized by daily temperature fluctuations of up to 7 °C, it was expected that 4 °C daily temperature variability would benefit their ability to cope with heat stress^12^. Especially in the weeks before temperatures started exceeding their native maximum summer temperature threshold, daily temperature maxima in the variable treatment should have triggered protective sub-bleaching stress^47^. However, this was not the case. Although several analyses have reported largely beneficial effects of temperature variability on coral heat tolerance^23,24^, some studies have also found detrimental impacts^48^, consistent with our findings here. Furthermore, acclimation to variable temperatures failed to improve thermal tolerance in a recent study, independent of the corals’ environmental history^49^. We suggest that some of these mechanisms may be taxon-specific, with heat-sensitive taxa such as *Acropora* typically benefitting more from thermal variability than heat-resistant taxa such as massive *Porites*^49^. In addition, in reef habitats associated with high thermal variability (e.g. tide pools or back reef), temperature usually co-varies with multiple other parameters such as light and flow, thus further complicating their combined impacts on heat tolerance. In the Kimberley, it is likely that highly variable light levels associated with tidal dynamics and high turbidity^29^ may increase the beneficial effect of temperature fluctuations on coral heat tolerance in their native environment but this was not simulated in our experiment.

In summary, we provide long-term experimental evidence that corals with high stress tolerance yet living close to their upper thermal limits have bleaching threshold temperatures that remain unchanged despite long-term acclimation to both warmer and cooler temperatures. While this apparent rigidity of thermal thresholds highlights their increasing vulnerability to a rapidly changing climate, it also suggests that these corals could potentially play an important role in facilitating coral reef survival via exchange of heat-tolerant alleles with more thermally sensitive reef regions—provided that their superior heat tolerance has indeed a strong genetic and thus heritable basis, as observed in other corals^3,15,37,50^. However, models show that the natural exchange of heat-tolerant alleles through dispersal of larvae may not occur at rates fast enough to keep pace with rapid climate change^5,16^, whereas human-assisted translocation could potentially enhance the heat tolerance of local populations at a rate sufficient to keep up with climate change^16^. Therefore, new interventions may be required to support coral reef persistence under higher-emission climate scenarios despite the considerable risks and scalability challenges of such approaches^17,18,38^. The high natural stress resistance of Kimberley corals and their ability to tolerate prolonged aerial exposure make these corals resistant to handling stress (V. Schoepf, pers. obs.), which would be advantageous for human-assisted translocation. Given their ability to maintain both high heat tolerance and health under much cooler temperatures, our study therefore highlights that corals from naturally extreme, thermally variable reef environments have significant potential for new interventions to conserve and manage coral reefs threatened by climate change.

## Methods

### Coral collection

We used the common, reef-building coral species *A. aspera* from the macrotidal Kimberley region in NW Australia as study organism (Fig. 1b). This species is among the dominant coral species on shallow Kimberley coral reefs^51^ and the dominant species at our study site^26^. Intertidal populations of this species are known to have a superior heat tolerance promoted by the extreme environmental conditions in this region and are dominated by symbionts from the genus *Cladocopium* (previously clade C)^12,46^. Eleven visibly healthy colonies were collected in April 2016 from the intertidal at Shell Island, Cygnet Bay (16°28′45.8”S, 123°2′41.3”E). Colonies were collected at least 10 m apart to avoid collecting clones. Relevant permits were obtained from the Department of Fisheries (exemption no. 2549, date of issue 3 March 2015). This site features a tidal range of up to 8 m (Fig. 1b); thus intertidal corals have a naturally high stress tolerance because they regularly experience prolonged aerial exposure, high light levels and extreme daily temperature fluctuations of up to 7 °C, with short-term maxima of up to 37 °C^12,29^. Monthly average temperatures range from ~25 to 31 °C (Fig. 1a, Supplementary Table 1), and the bleaching threshold was experimentally established to be ~32 °C^12^.

Colonies were live-shipped to the University of Western Australia and maintained in indoor, flow-through aquaria at the Watermans Bay seawater facility at ~29 °C to facilitate recovery and acclimation to tank conditions. Temperatures were kept constant within ~1 °C. From mid-June until the end of July 2016, temperatures were adjusted twice a month to mimic seasonal temperatures at the collection site (Supplementary Table 1). Light was provided on a 12:12 h light:dark cycle, following a natural diurnal light cycle with gradual increases up to 560 μmol m^−2^ s^−1^ at noon. Corals were fed twice a week with live brine shrimp. Further details on the feeding regime and mesocosm tank set-up are given below. In July 2016, each colony was fragmented into 6 pieces of 5–10 cm which were glued onto pre-labelled plastic tiles. Coral fragments were allowed to recover for ~2 weeks prior to the start of the experiment at the beginning of August 2016. Ethics approval is not required for corals.

### Mesocosm tank set-up

Coral fragments were maintained in 55-L transparent plastic tanks where seawater was being replaced at a rate of ~0.5 L min^−1^. Water motion was provided using a submersible pump (Macro Aqua, 3000 L h^−1^) connected to a flow controller set at the highest speed. Temperature was maintained using titanium heaters (WeiPro, 500 or 1000 Watt) and controlled via the ApexFusion software (Neptune Systems). The Apex temperature probes were calibrated 1–2 times a week using a high-precision thermometer (Fisher Scientific Traceable). Light was provided on a 12:12 h light:dark cycle (06:00–18:00 hours) using 150 W LED lights (Ledzeal S150 Plus) with custom-designed LED arrangements and colours to ensure a light spectrum similar to shallow tropical reef environments. The lights were programmed to follow a natural diurnal light cycle, with gradual increases up to 560 μmol m^−2^ s^−1^ at noon (measured using an Apogee MQ-200 cosine-corrected planar PAR-meter). Relatively high maximum light levels were chosen because intertidal Kimberley corals regularly experience high light levels depending on tidal elevation, water clarity and cloud cover (up to ~2000 µmol m^−2^ s^−1^)^29^; however, these extreme light levels are only experienced short term during spring low tide, therefore intermediate levels were used for daily exposure in this study. The incoming seawater was pumped from 12 m depth and filtered through three sand filters (~20 μm nominal size). Corals were fed twice a week with live brine shrimp. Approximately 2.5 g of brine shrimp eggs were hatched, and the stock solution with live nauplii was then equally divided among all tanks. HOBO v2 temperature loggers were deployed in each tank and continuously recorded seawater temperature every 5 min.

### Temperature acclimation phase

From 1 August 2016 through mid-April 2017, coral fragments were exposed to one of three seasonal temperature treatments (Fig. 1a): (1) native Kimberley temperatures (native control), (2) mid-century Kimberley temperatures (warming; =control + 1 °C), and (3) temperatures representative of Ningaloo Reef in Western Australia (4 °C cooler reef; =control − 4 °C), which is located 1200 km southwest of the Kimberley collection site and has much cooler seasonal temperatures as well as much lower daily temperature variability^29^ (22–27.5 °C; Supplementary Tables 1 and 2). Temperatures in the 4 °C cooler reef treatments were gradually lowered from 1 August until 1 September (−0.5 °C every 5 days) to prevent cold shocking the corals. To explore the role of temperature variability in promoting acclimation, the three seasonal temperature treatments were crossed with two daily temperature variability regimes: constant daily temperatures or 4 °C daily temperature variability (Supplementary Fig. 1). This resulted in a total of six treatments, with two replicate tanks per treatment. Although Ningaloo Reef represents a thermally more stable environment than the Kimberley^29^, 4 °C daily temperature variability was nevertheless combined with the cooler temperature treatment to (1) have a fully factorial design and (2) to assess whether temperature variability influences the ability to maintain heat tolerance under long-term cold acclimation. Temperatures were adjusted twice per month to follow the seasonal temperature treatments. One fragment from each of the 11 parent colonies was present in the six temperature treatments (*n* = 11 per treatment), and fragments were randomly assigned to replicate tanks.

By January 2017, corals in the warming treatments (particularly in the variable regime) began to show signs of paling and progressive bleaching (Supplementary Fig. 2a, b) as temperatures started exceeding the MMM temperatures at their collection site. This was accompanied by significant declines in photochemical efficiency and substantially reduced *P*/*R* ratios (Figs 2, 4), indicating severe heat stress and the risk of considerable mortality due to rapid tissue necrosis typical for *Acropora* corals when exposed to prolonged heat stress^12^. Therefore, temperatures in these treatments were lowered by 2 °C on 3 February 2017 to facilitate recovery and maintained at native control − 1 °C seasonal temperatures until the start of the heat stress test in mid-May 2017 (Fig. 1a, Supplementary Table 2). Temperatures were lowered in both constant and variable treatments to enable assessment of how daily temperature variability affects warm acclimation.

Almost all experimental corals survived the acclimation phase, with the exception of one coral in the variable warming treatment, which died after 6 months in February 2017. A further six corals in the constant warming treatment died on 24 March 2017 after a heater malfunctioned. The remaining five corals in this treatment were then divided between the two replicate tanks.

Coral acclimation capacity was assessed based on a number of key health traits related to both coral host and algal symbiont. They included visual coral health using the CoralWatch® Coral Health Chart^52^; calcification rates using the buoyant weight technique^53^; the ratio of photosynthesis to respiration (daily *P*/*R* ratio), indicating net autotrophy or net heterotrophy; and the maximum quantum yield of photosystem II (Fv/Fm), which is an indicator of photochemical efficiency. During the heat stress test, we further used the photochemical efficiency as a highly sensitive indicator of changes in heat tolerance^30^ due to long-term exposure to warmer/cooler temperatures. Further details are given below.

### Heat stress test

In order to assess the influence of long-term cold/warm acclimation and daily temperature variability on coral heat tolerance, a heat stress test was conducted at the end of the 9-month acclimation phase. Each of the treatments was split into a (i) control treatment maintained at ambient temperatures (*n* = 5) and (ii) heat stress treatment where temperatures were gradually increased to the known bleaching threshold of ~32 °C (*n* = 6) (Fig. 1a, b). Parent colonies had randomly been assigned numbers prior to the start of the overall experiment, and at this stage, colonies #1–5 were assigned to the control treatment, whereas colonies #6–11 were assigned to the heat stress treatment. A higher sample size was chosen for the stress treatment to account for potentially higher within-treatment variability. Owing to logistical constraints associated with maintaining corals during these challenging long-term experiments, it was not possible to double the number of tanks to have full tank replication during the heat stress test; however, full tank replication was achieved for the critical 9-month acclimation phase. A heating rate of 1 °C per week was used for all heat stress treatments; thus temperature ramp up in the 4 °C cooler tanks already started on 18 April 2017, whereas the ramp up for the warming and native control treatments started on 28 April and 5 May, respectively. During the ramp up, ambient temperatures were ~26.7 and ~30.1 °C for the −4 °C and warming/native treatments, respectively (Supplementary Table 2). The same ambient and heat stress temperatures were used for both warming and native treatments because the warming corals had already experienced bleaching in January 2017.

On 17 May 2017, all heat stress tanks had reached the target temperature of 32 °C (day 1) and the heat stress test commenced (Fig. 1a, b). This temperature was sustained for 6 days and then elevated by 0.5 °C to increase thermal stress for a further 7 days (13 days of total heat stress), resulting in an average temperature of ~32.4 °C in the heat stress tanks (Supplementary Table 2), which is ~1.6 °C above the MMM temperatures at the collection site^12^. Ambient temperatures during the heat stress test were ~26.4 and ~29.9 °C for the −4 °C and warming/native treatments, respectively (Fig. 1a, Supplementary Table 2). The heat stress test ended on 30 May 2017, and temperature in the heat stress tanks was returned to the respective ambient, seasonal temperatures.

### Photo-physiology

Photo-physiological performance was assessed monthly for the first 6 months, then weekly during month 7 when corals in the warming treatment started to bleach and then 1–2 times per month until the start of the heat stress test. During the heat stress test, measurements were conducted daily for 13 days. Fv/Fm was measured 1 h after lights turned off to assess the photochemical efficiency in the dark-adapted state. Measurements were made using a diving-PAM underwater fluorometer (Walz, Germany) with the following settings: measuring light intensity = 3, saturation pulse intensity = 12, saturation pulse width = 0.8 s, gain = 5, and damping = 2. Measurements were made at a constant distance of 2 mm from the coral tissue, approximately 2–3 cm below the tip. Variable and constant temperature treatments were assessed at the same temperature (i.e. temperatures in variable tanks were set to the same temperature as in the constant tanks from 18:00 to 21:00 hours for Fv/Fm—see Supplementary Fig. 1).

### Health score

Coral health was determined using the CoralWatch® Coral Health Chart^52^ monthly for the first 6 months, then twice a month until the start of the heat stress test. During the heat stress test, readings were taken at the beginning and end of the heat stress test. Corals were scored on the upper surface of the branches. Although visual assessment of coral health is less sensitive than quantification of symbiont density and/or chlorophyll *a* content, we chose this method because it is non-destructive and thus allowed for repeated measurements over the course of this long-term experiment. The brightness/saturation scale of the Coral Health Chart was developed and rigorously calibrated using analyses of symbiont density and chlorophyll *a* content, such that a change of two units in brightness indicates a significant change in symbiont density and chlorophyll *a* content and thus the bleaching state of corals^52^.

### Photosynthesis and respiration

Whole-fragment net photosynthesis (*P*) and respiration (*R*) rates were determined over ~10 days after 3 and 6 months of acclimation and immediately following the heat stress test. Variable and constant temperature treatments were assessed at the same temperature. For measurements conducted at the end of the heat stress test (i.e. after heat stress ended), heat-stressed corals were incubated prior to ambient corals to minimize recovery effects. Both ambient and heated tanks were maintained at the same temperatures (~30.0 °C) to avoid confounding effects of temperature on *P* and *R* rates. Only in the −4 °C treatments were corals incubated at different temperatures (ambient tanks: ~26.4 °C, heated tanks: ~30.0 °C), as decreasing temperatures in the heated tanks from ~32.4 to ~26.4 °C would have likely created additional stress for these corals.

*P* and *R* rates were determined via oxygen production and consumption, respectively, by incubating corals in sealed, clear plastic chambers (1.0 L at the first two time points, 1.75 L at the end of the heat stress test). Chambers were placed in a water bath with temperature control to maintain constant temperatures. Turbulent water motion inside the chambers was achieved by placing the chambers on a submersible magnetic stirring plate (2Mag MIXdrive 6, John Morris Scientific, stir bar speed of 500 rpm). Each incubation round (up to five corals) had one control incubation where a clean coral skeleton on a tile (similar to the one used for the experimental corals) was placed inside the chamber. For light incubations, corals were exposed to constant light intensity of 560 μmol m^−2^ s^−1^ to match maximum light intensities in the culture tanks. Two rounds of light incubations were conducted between ~10:00 and 14:00 hours, whereas dark incubations were conducted between ~08:00–10:00 and ~16:00–18:00 hours. Corals were either dark-adapted overnight or for 1.5 h prior to the dark incubations (from ~14:30 to 16:00 hours). Incubation duration varied from 50 min to 2 h depending on the size of the coral fragment to achieve a ~15% change in O_2_ saturation. At the beginning and end of each incubation, oxygen (Orion Star A323 RDO/DO meter, Thermo Scientific), salinity (YSI 85), pH and temperature (Schott handylab pH12) were measured. The volume of the incubation seawater within the chambers was measured by pouring the seawater into a graduated cylinder after all measurements were completed. Hourly oxygen data were converted from percentage of O_2_ saturation to μmol L^−1^ seawater using the equations of Garcia and Gordon^54^ and normalized to surface area (see below). Given the 12 h:12 h light:dark regime, *P*/*R* ratios were calculated as 12 h of gross *P* ( = net *P* + *R*) divided by 24 h of *R*.

### Calcification

Area-normalized calcification rates were determined using the buoyant weight technique^53^ at monthly time intervals and at the beginning and end of the 13-day heat stress test. The wet weight of the coral (minus the combined weight of the tile and epoxy) was converted to dry weight using the density of seawater based on measurements of salinity and temperature and the known density of aragonite (2.93 g cm^−3^). The difference in dry weight (mg) of the coral between two time points was then calculated and divided by both the number of days (days) between the two time points and the surface area (cm^2^) to obtain daily area-normalized calcification rate in mg day^−1^ cm^−2^. Following Foster et al.^55^, the surface area was calculated as the average of the surface area values at the two time points. Surface area was calculated using the relationship between coral skeletal mass (dry weight) and surface area determined via computed tomographic scans of skeletons of various sizes from the same coral species from the same location (Supplementary Fig. 3).

### Statistical analyses

Generalized linear mixed model (GLMM) analysis was used to analyse the effects of temperature (3 levels: native control, warming, 4 °C cooler reef), daily temperature variability (2 levels: constant, variable), and time (various levels) on the response variables. For analyses related to the heat stress test, an additional factor was included in the analyses to assess the effect of heat stress (2 levels: ambient, heat stress) on the response variables. In all analyses with time as a factor, individuals were thus measured repeatedly. Parent colony was included as a random factor, except when the estimated *G* matrix was not positive definite, indicating that the variance component of the random factor was estimated to be zero and could/should be removed from the model (i.e. photosynthesis during the acclimation phase)^56^. Residuals were visually inspected to check assumptions associated with GLMM analysis. Tukey adjusted *p* values were used for post hoc tests when main effects were significant. When a significant interaction was observed, multiple pair-wise comparisons were conducted using Tukey adjusted *p* values. Analyses were performed using Proc Glimmix in the SAS software, version 9.4 of the SAS System for Windows. *p* Values <0.05 were considered significant.

### Reporting summary

Further information on research design is available in the Nature Research Reporting Summary linked to this article.

### Supplementary information

Supplementary Information

Reporting Summary

Supplementary Data 1

### Source data

Source Data

## Data Availability

All data generated or analysed during this study are included in this published article and its source data files. The source data underlying Figs. 1–6, Supplementary Figs. 1–3 and Supplementary Tables 3–4 are provided as a Source Data file. Additional temperature data underlying the data presented in Fig. 1 are provided as Supplementary Data 1 file. The source data underlying Supplementary Table 1 were obtained online (see Source Data file for more info).
