# Author Correction: Stress-resistant corals may not acclimatize to ocean warming but maintain heat tolerance under cooler temperatures

**DOI:** 10.1038/s41467-023-41902-6

**Published:** 2023-10-04

**Authors:** Verena Schoepf, Steven A. Carrion, Svenja M. Pfeifer, Melissa Naugle, Laurence Dugal, Jennifer Bruyn, Malcolm T. McCulloch

**Affiliations:** 1 Oceans Graduate School and UWA Oceans Institute, The University of Western Australia, 35 Stirling Highway, Perth, WA 6009 Australia; 2 ARC Centre of Excellence for Coral Reef Studies, The University of Western Australia, 35 Stirling Highway, Perth, WA 6009 Australia; 3 School of Geosciences, University of Edinburgh, James Hutton Road, Edinburgh, EH9 3FE UK; 4 Department of Biology, Heinrich-Heine-Universität Düsseldorf, Universitätsstrasse 1, 40225 Düsseldorf, Germany

Correction to: *Nature Communications* 10.1038/s41467-019-12065-0, published online 17 September 2019

The original version of this Article contained errors in the Results section, in Figs. 4 and 6 and in the Source Data. Due to an error in the equation used to calculate the photosynthesis to respiration ratios (*P*/*R*), the *P*/*R* ratios were higher than they should have been. The relative differences between treatment groups and, thus, the outcomes of the statistical tests were not affected. The affected sentences in the Results incorrectly read “45% lower photosynthesis to respiration” and “−29 to −40%”, “up to −76% vs. up to −50%”. The correct versions state “35% lower photosynthesis to respiration”, “−23 to −30%”, “up to −62% vs. up to −36%”.

Corrected Fig. 4


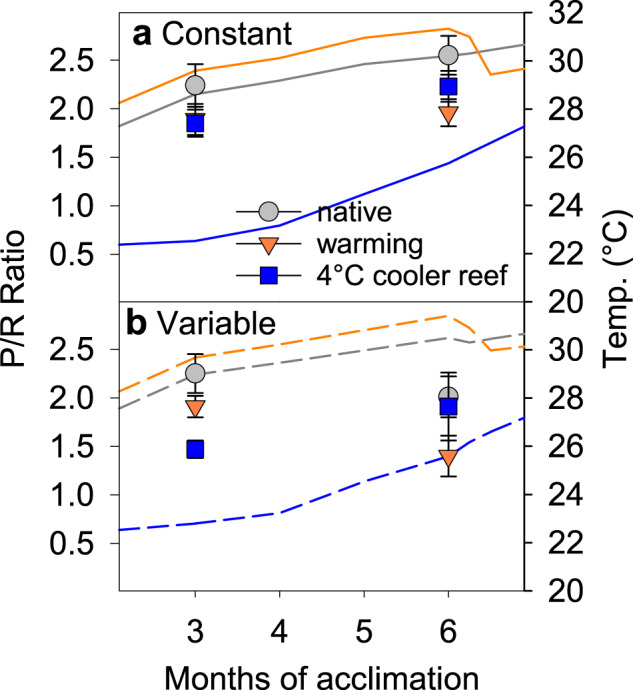


Corrected Fig. 6


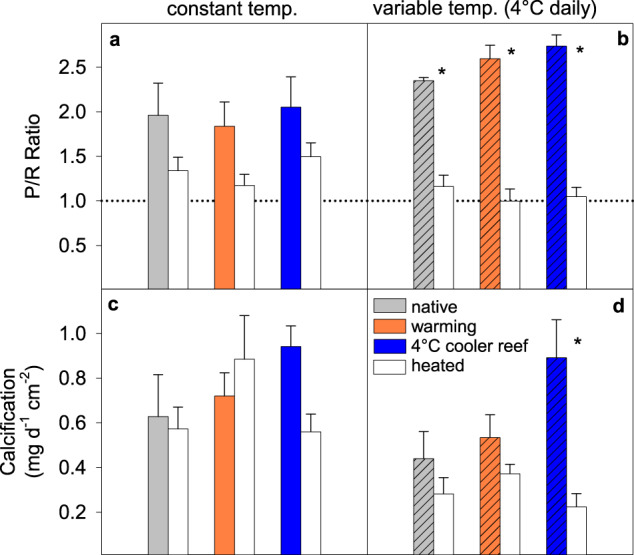


This has been corrected in both the PDF and HTML versions of the Article. The correct versions of Figs. 4 and 6 and of the Source Data file, which replace the previous incorrect versions, are attached.

### Supplementary information

Updated Source Data

