# Supplementary material for: Stress-resistant corals may not acclimatize to ocean warming but maintain heat tolerance under cooler temperatures
Source: Nat Commun. 2019 Sep 17;10:4031. doi: 10.1038/s41467-019-12065-0 (PMC6748961; doi:10.1038/s41467-019-12065-0)
Supplement: Supplementary file 1 — Supplementary Information [file 41467_2019_12065_MOESM1_ESM.pdf]

Supplementary Information

Stress-resistant corals may not acclimatize to ocean warming but maintain heat tolerance under cooler temperatures

Schoepf et al.

Supplementary Tables

Supplementary Table 1. Monthly seasonal sea surface temperature (SST) data for the Kimberley region and Ningaloo Reef in Western Australia. The SST data served as target temperatures for the acclimation phase of the experiment (see Table S2). Data sourced from the U.S. National Oceanic and Atmospheric Administration (NOAA), Coral Reef Watch, 5-km virtual stations "North Western Australia" and "Ningaloo" (version 2).

Month	Experimental month	SST Kimberley (°C)	SST Ningaloo (°C)	SST Difference (°C)
January	6	30.1	25.1	5.0
February	7	30.2	27.0	3.2
March	8	30.6	27.4	3.2
April	Temp. ramp up	30.8	27.4	3.4
May	Heat stress test	29.7	26.3	3.3
June		27.9	24.8	3.1
July		26.1	23.4	2.6
August	1	25.6	22.4	3.3
September	2	26.5	22.1	4.4
October	3	28.0	22.2	5.8
November	4	29.3	23.0	6.4
December	5	29.6	23.9	5.7
Average		28.7	24.6	4.1

Supplementary Table 2. Average water temperatures during the (a) 9-month acclimation phase and the subsequent (b) temperature ramp-up and (c) 13-day heat stress test in treatments with constant daily temperature and with 4°C daily temperature variability. Averages are shown ±S.D. Sample size (i.e. the number of temperature logger measurements) was 8064-8928 for monthly averages, 8262-8352 for the temperature ramp up phase, and 3728-3744 for the heat stress phase. The raw data are provided in Supplementary Data 1.

	**Native Kimberley**		**Warming (Native Kimberley +1°C)**		**4°C cooler reef (Ningaloo)**	
	**constant**	**variable**	**constant**	**variable**	**constant**	**variable**
**(a) Acclimation phase**						
Aug. '16	25.55 ± 0.38[1]	26.31 ± 1.26	27.21 ± 0.35	26.94 ± 1.25	24.28 ± 0.91[2]	24.27 ± 1.54[2]
Sept. '16	27.14 ± 0.51	27.41 ± 1.33	28.11 ± 0.51	28.11 ± 1.32	22.36 ± 0.23	22.50 ± 1.32
Oct. '16	28.62 ± 0.41	28.97 ± 1.22	29.59 ± 0.38	29.68 ± 1.24	22.53 ± 0.26	22.80 ± 1.30
Nov. '16	29.18 ± 0.69	29.46 ± 1.30	30.11 ± 0.47	30.22 ± 1.31	23.16 ± 0.79	23.23 ± 1.45
Dec. '16	29.86 ± 0.25	29.97 ± 1.18	30.95 ± 0.29	30.81 ± 1.17	24.47 ± 0.42	24.54 ± 1.32
Jan. '17	30.21 ± 0.46	30.49 ± 1.18	31.33 ± 0.28	31.41 ± 1.19	25.75 ± 0.56	25.58 ± 1.44
Feb. '17	30.72 ± 0.23	30.71 ± 1.19	29.72 ± 0.59	30.18 ± 1.31	27.44 ± 0.33	27.33 ± 1.36
Mar. '17	30.71 ± 0.28	31.06 ± 1.19	29.61 ± 0.76	30.05 ± 1.19	27.60 ± 0.22	27.83 ± 1.22
1-17 April '17	30.69 ± 0.31	30.90 ± 1.20	29.67 ± 0.27	29.97 ± 1.20	27.61 ± 0.22	27.59 ± 1.22
**(b) Temperature ramp-up** (18 April – 16 May '17)[3]						
Ambient	30.41 ± 0.45	30.26 ± 1.30	29.84 ± 0.47	29.97 ± 1.22	26.63 ± 0.41	26.73 ± 1.27
Heated	30.45 ± 0.69	30.99 ± 1.22	30.39 ± 1.04	30.47 ± 1.49	30.02 ± 1.29	30.00 ± 1.78
**(c) Heat stress test** (17-29 May '17)						
Ambient	29.81 ± 0.37	30.00 ± 1.17	29.92 ± 0.34	29.90 ± 1.14	26.37 ± 0.21	26.50 ± 1.19
Heated	32.28 ± 0.38	32.37 ± 1.16	32.50 ± 0.43	32.42 ± 1.17	32.31 ± 0.41	32.41 ± 1.14

[1]Note that values were lower than target values due to temperature probe issues

[2]Temperatures were gradually lowered over 4 weeks to prevent cold-shocking corals

[3]Note that average temperature and SD differ across heated tanks because the temperature ramp up started at different time points and seasonal baseline temperatures for the various temperature treatments (see Methods for more details).

Supplementary Table 3. Results from generalized linear mixed models to test for the effects of temperature (temp.), temperature variability (var.) and time on the photochemical efficiency (Fv/Fm), calcification rate (calc.), P/R ratios and health chart scores of *Acropora aspera* during the acclimation phase. Post hoc Tukey tests results are given when main effects (but no interaction terms) were significant. Effects with p-values ≤0.05 are highlighted in bold. Num df = numerator degrees of freedom, den df = denominator degrees of freedom. Var. = variable. The data underlying these statistical analyses are the same as for Figs 2-4 and Supplementary Fig 2.

Var.	Effect	Num df	Den df	*F*-statistic	*p*-value	Tukey
**Fv/Fm**	Temp	2	749	48.57	**<0.0001**	
	Time	13	749	49.39	**<0.0001**	
	Temp * time	23	749	9.36	**<0.0001**	See text
	Var	1	749	113.44	**<0.0001**	
	Temp * var	2	749	9.33	**<0.0001**	See text
	Var * time	13	749	8.21	**<0.0001**	See text
	Temp * var * time	23	749	1.53	0.0532	
**Calc.**	Temp	2	511	5.34	**0.0051**	
	Time	8	511	28.60	**<0.0001**	See text
	Temp * time	16	511	4.39	**<0.0001**	See text
	Var	1	511	14.43	**0.0002**	
	Temp * var	2	511	9.20	**0.0001**	See text
	Var * time	8	511	3.12	**0.0019**	See text
	Temp * var * time	16	511	1.63	0.0568	
**P/R**	Temp	2	38	7.81	**0.0014**	native > warming = 4°C cooler reef
	Time	1	38	0.29	0.5925	
	Temp * time	2	38	3.01	0.0613	
	Var	1	38	6.27	**0.0167**	Constant > variable
	Temp * var	2	38	0.02	0.9767	
	Var * time	1	38	2.85	0.0997	
	Temp * var * time	2	38	0.95	0.3959	
**Health**	Temp	2	672	6.36	**0.0018**	
	Time	11	672	45.68	**<0.0001**	
	Temp * time	21	672	15.22	**<0.0001**	See text
	Var	1	672	13.05	**0.0003**	
	Temp * var	2	672	13.53	**<0.0001**	See text
	Var * time	11	672	2.42	**0.0060**	See text
	Temp * var * time	21	672	1.16	0.2817	

Supplementary Table 4. Results from generalized linear mixed models to test for the effects of temperature (temp.), temperature variability (var.), heat stress and time (if applicable) on the photochemical efficiency (Fv/Fm), calcification rate (calc.), P/R ratios and health chart scores of *Acropora aspera* during the heat stress test. Calcification, P/R and coral health were only measured at the end of the heat stress test. Post hoc Tukey tests results are given when main effects (but no interaction terms) were significant. Effects with p-values ≤0.05 are highlighted in bold. Num df = numerator degrees of freedom, den df = denominator degrees of freedom. Var. = variable. The data underlying these statistical analyses are the same as for Figs 5-6 and Supplementary Fig 2.

Var.	Effect	Num df	Den df	*F*-statistic	*p*-value	Tukey
**Fv/Fm**	Temp	2	645	32.51	**<0.0001**	
	Var	1	645	50.71	**<0.0001**	
	Temp * var	2	645	4.78	**0.0087**	See text
	Heat	1	645	86.65	**<0.0001**	
	Temp * heat	2	645	7.89	**0.0004**	See text
	Var * heat	1	645	15.90	**<0.0001**	See text
	Temp * var * heat	2	645	1.57	0.2092	
	Time	13	645	38.61	**<0.0001**	See text
	Temp * time	26	645	0.94	0.5442	
	Var * time	13	645	0.85	0.6087	
	Temp * var * time	26	645	0.41	0.9962	
	Heat * time	13	645	28.29	**<0.0001**	See text
	Temp * heat * time	26	645	0.87	0.6484	
	Var * heat * time	13	645	1.26	0.2314	
	Temp * var * heat * time	26	645	0.28	0.9999	
**Calc.**	Temp	2	37	7.14	**0.0024**	
	Var	1	37	27.60	**<0.0001**	Constant > variable
	Temp * var	2	37	0.86	0.4333	
	Heat	1	37	5.51	**0.0244**	
	Temp * heat	2	37	9.53	**0.0005**	See text
	Var * heat	1	37	2.83	0.1009	
	Temp * var * heat	2	37	0.30	0.7454	
**P/R**	Temp	2	24	0.89	0.4236	
	Var	1	24	2.07	0.1629	
	Temp * var	2	24	0.22	0.8015	
	Heat	1	24	64.18	**<0.0001**	

	Temp * heat	2	24	0.40	0.6739	
	Var * heat	1	24	12.26	**0.0018**	See text
	Temp * var * heat	2	24	0.49	0.6215	
**Health**	Temp	2	37	0.92	0.4067	
	Var	1	37	0.15	0.6965	
	Temp * var	2	37	2.28	0.1163	
	Heat	1	37	261.52	**<0.0001**	
	Temp * heat	2	37	6.56	**0.0036**	See text
	Var * heat	1	37	6.87	**0.0126**	See text
	Temp * var * heat	2	37	4.26	**0.0216**	See text

Supplementary Figures

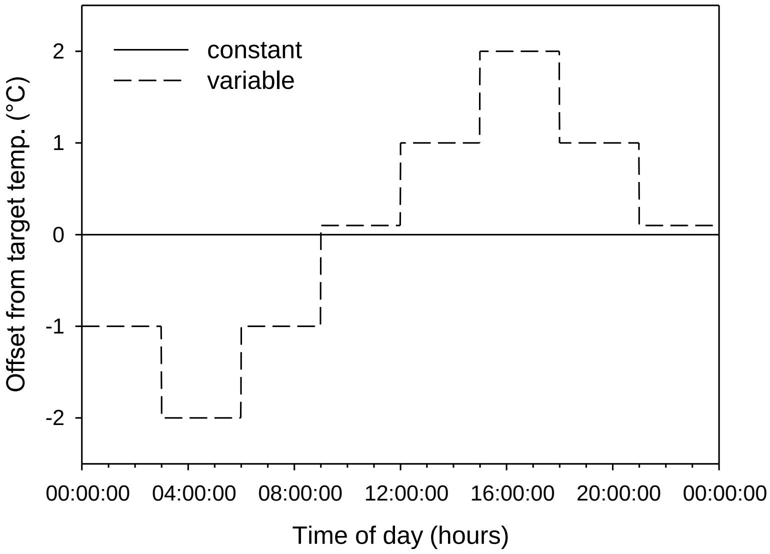

Supplementary Figure 1: Schematic of the temperature regimes in treatments with constant daily temperature versus 4°C daily variability.

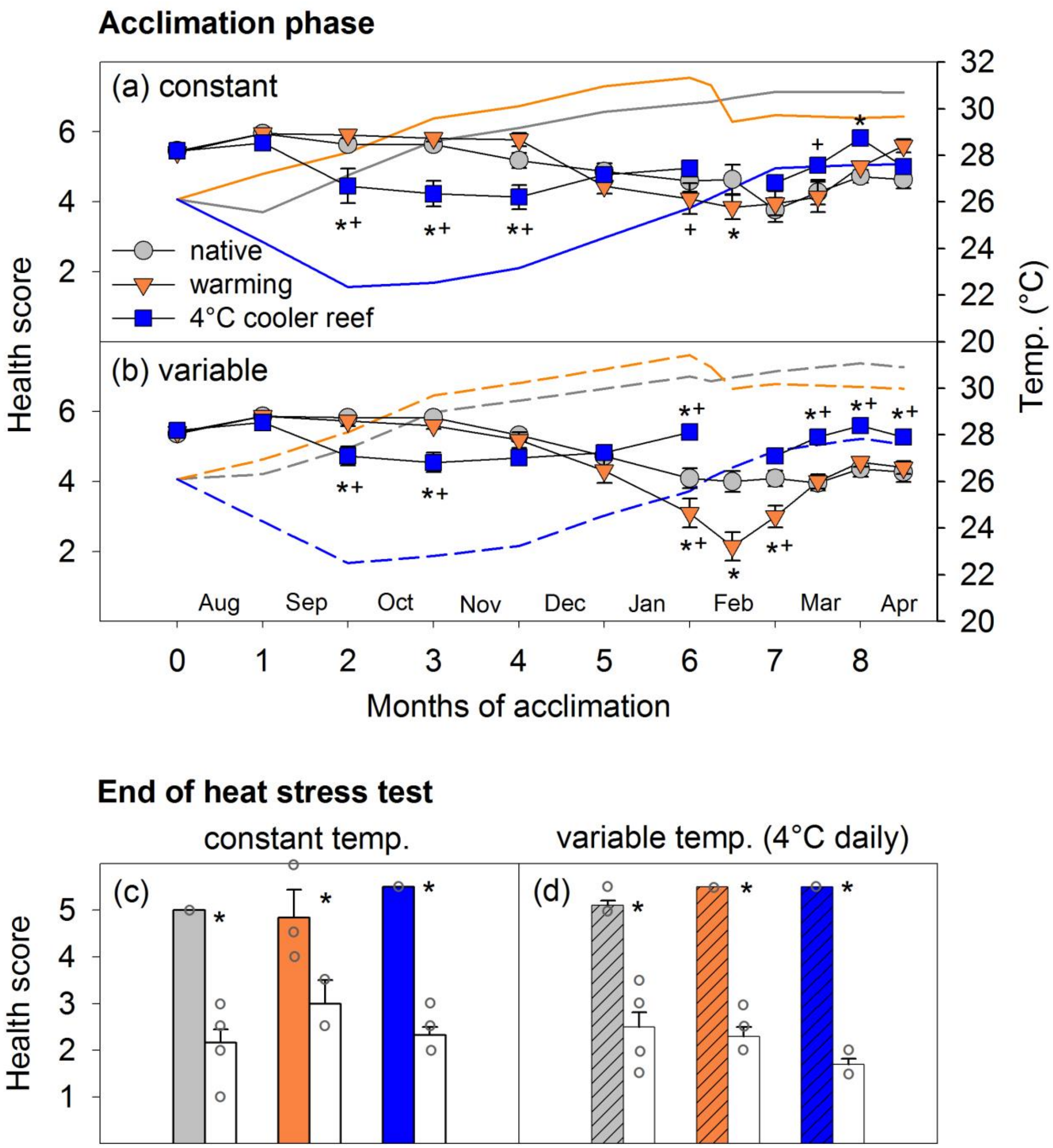
Acclimation phase
(a) constant
native
warming
4°C cooler reef
(b) variable
Health score
Temp. (°C)
Aug
Sep
Oct
Nov
Dec
Jan
Feb
Mar
Apr
Months of acclimation
End of heat stress test
constant temp.
variable temp. (4°C daily)
(c)
(d)
Health score

Supplementary Figure 2. Coral health chart score during the acclimation phase and at the end of the heat stress test. *Acropora aspera* corals were maintained under **(a, c)** constant daily temperatures or **(b, d)** 4°C daily temperature variability. Mean ± 1S.E.M. is shown. For bar charts, grey circles indicate individual data points. Higher values indicate greater coral health. In panels a and b, solid and dashed lines represent monthly tank temperatures for each treatment (Table S2). Asterisks indicate a significant difference from the native (control) treatment, whereas + indicates a significant difference between the warming and 4°C cooler reef treatments ($p<0.05$; Tukey posthoc tests). Note that 4°C cooler reef corals were not assessed at month 6.5 In panels c and d, open bars represent heat-stressed corals at each treatment, whereas asterisks indicate a significant difference between ambient and heat-stressed corals at each treatment ($p<0.05$; Tukey posthoc tests). Source data are provided as a Source Data file.

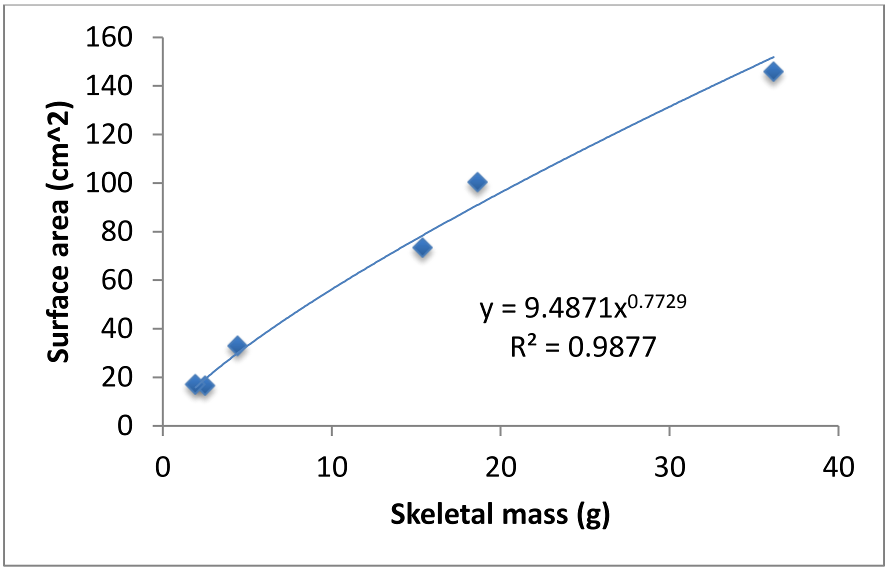

Supplementary Figure 3. Relationship between coral skeletal mass and CT-based surface area. Corals of the species *Acropora aspera* from Cygnet Bay, Kimberley region, NW Australia, were used for this calibration. Source data are provided as a Source Data file.
