# Supplementary material for: Stress-resistant corals may not acclimatize to ocean warming but maintain heat tolerance under cooler temperatures
Source: Nat Commun. 2019 Sep 17;10:4031. doi: 10.1038/s41467-019-12065-0 (PMC6748961; doi:10.1038/s41467-019-12065-0)
Supplement: Supplementary file 2 — Reporting Summary [file 41467_2019_12065_MOESM2_ESM.pdf]

nature research

Corresponding author(s): Verena Schoepf

Last updated by author(s): 16-04-2019

Reporting Summary

Nature Research wishes to improve the reproducibility of the work that we publish. This form provides structure for consistency and transparency in reporting. For further information on Nature Research policies, see Authors & Referees and the Editorial Policy Checklist.

Statistics

For all statistical analyses, confirm that the following items are present in the figure legend, table legend, main text, or Methods section.

n/a	Confirmed	
☐	☒	The exact sample size (n) for each experimental group/condition, given as a discrete number and unit of measurement
☐	☒	A statement on whether measurements were taken from distinct samples or whether the same sample was measured repeatedly
☐	☒	The statistical test(s) used AND whether they are one- or two-sided<br>*Only common tests should be described solely by name; describe more complex techniques in the Methods section.*
☐	☒	A description of all covariates tested
☐	☒	A description of any assumptions or corrections, such as tests of normality and adjustment for multiple comparisons
☐	☒	A full description of the statistical parameters including central tendency (e.g. means) or other basic estimates (e.g. regression coefficient) AND variation (e.g. standard deviation) or associated estimates of uncertainty (e.g. confidence intervals)
☐	☒	For null hypothesis testing, the test statistic (e.g. F, t, r) with confidence intervals, effect sizes, degrees of freedom and P value noted<br>*Give P values as exact values whenever suitable.*
☒	☐	For Bayesian analysis, information on the choice of priors and Markov chain Monte Carlo settings
☐	☒	For hierarchical and complex designs, identification of the appropriate level for tests and full reporting of outcomes
☒	☐	Estimates of effect sizes (e.g. Cohen's d, Pearson's r), indicating how they were calculated

Our web collection on statistics for biologists contains articles on many of the points above.

Software and code

Policy information about availability of computer code

Data collection	Analyses were performed using Proc Glimmix in SAS software, version 9.4 of the SAS System for Windows. (this is stated in the main text)
Data analysis	Analyses were performed using Proc Glimmix in SAS software, version 9.4 of the SAS System for Windows. (this is stated in the main text)

For manuscripts utilizing custom algorithms or software that are central to the research but not yet described in published literature, software must be made available to editors/reviewers. We strongly encourage code deposition in a community repository (e.g. GitHub). See the Nature Research guidelines for submitting code & software for further information.

Data

Policy information about availability of data

All manuscripts must include a data availability statement. This statement should provide the following information, where applicable:

- Accession codes, unique identifiers, or web links for publicly available datasets
- A list of figures that have associated raw data
- A description of any restrictions on data availability

All data generated or analysed during this study are included in this published article and its supplementary information files (see source data file and dataset for Supplementary Table 2).

Field-specific reporting

Please select the one below that is the best fit for your research. If you are not sure, read the appropriate sections before making your selection.

☐ Life sciences ☐ Behavioural & social sciences ☒ Ecological, evolutionary & environmental sciences

Ecological, evolutionary & environmental sciences study design

All studies must disclose on these points even when the disclosure is negative.

Study description	Using a long-term (~9 months) mesocosm experiment, we asked whether these corals can acclimate to ocean warming predicted to occur by mid-century (+1°C) when superimposed on their already thermally extreme native habitat over seasonal time scales. In parallel, we tested their potential for natural or human-assisted migration by simulating translocation from their hot, thermally variable reef in the Kimberley region to the much cooler, thermally stable Ningaloo Reef, a reef 1200 km southwest of the Kimberley region. To do so, we exposed them to 4°C cooler temperatures for 9 months to assess whether they could survive and maintain their high heat tolerance on cooler, thermally stable reefs. Finally, we explored the role of temperature variability in promoting acclimatization to warmer/cooler temperature regimes, as fluctuating temperatures often enhance coral heat tolerance.<br><br>From 1 August 2016 through mid-April 2017, coral fragments were exposed to one of three seasonal temperature treatments (Fig. 1a): representing (1) native Kimberley temperatures (native control), (2) mid-century Kimberley temperatures (warming; = control +1°C), and (3) temperatures representative of Ningaloo Reef in Western Australia (4°C cooler reef; = control -4°C), which is located 1200 km southwest of the Kimberley collection site and has much cooler seasonal temperatures as well as much lower daily temperature variability29 (22-27.5°C; Supplementary Tables S1, S2). To explore the role of temperature variability in promoting acclimation, the three seasonal temperature treatments were crossed with two daily temperature variability regimes: constant daily temperatures or 4°C daily temperature variability (Supplementary Figure 1). This resulted in a total of six treatments, with two replicate tanks per treatment. In order to assess the influence of long-term cold/warm acclimation and daily temperature variability on coral heat tolerance, a heat stress test was conducted at the end of the 9 months acclimation phase.
Research sample	We used the common, reef-building coral species Acropora aspera from the macrotidal Kimberley region in NW Australia as study organism (Fig. 1b). Intertidal populations of this species are known to have a superior heat tolerance promoted by the extreme environmental conditions in this region, despite beingand are dominated by generalist symbionts from the genus Cladocopium (previously clade C).
Sampling strategy	Eleven visibly healthy colonies were collected in April 2016 from the intertidal at Shell Island, Cygnet Bay (16°28’45.8”S, 123° 2’41.3”E). Colonies were collected at least 10 m apart to avoid collecting clones.
Data collection	Coral acclimation capacity was assessed based on a number of key health traits related to both coral host and algal symbiont. They included visual coral health using the CoralWatch® Coral Health Chart; calcification rates using the buoyant weight technique; the ratio of net photosynthesis to respiration (daily P/R ratio), indicating net autotrophy or net heterotrophy; and the maximum quantum yield of photosystem II or photochemical efficiency (Fv/Fm) . During the heat stress test, we further used the photochemical efficiency as a highly sensitive indicator of changes in heat tolerance due to long-term exposure to warmer/cooler temperatures. Data were collected by all co-authors except Malcolm McCulloch.
Timing and spatial scale	Photo-physiological performance was assessed monthly for the first 6 months, then weekly during month 7 when corals in the warming treatment starting to bleach, and then 1-2 times per month until the start of the heat stress test. During the heat stress test, measurements were conducted daily for 13 days.<br>Coral health was determined using the CoralWatch® Coral Health Chart monthly for the first 6 months, then twice a month until the start of the heat stress. During the heat stress test, readings were taken at the beginning and end of the heat stress test.<br>Whole-fragment net photosynthesis (P) and respiration (R) rates were determined over ~10 days after 3 and 6 months of acclimation, and immediately following the heat stress test.<br>Area-normalised calcification rates were determined using the buoyant weight technique at monthly time intervals and at the beginning and end of the 13-day heat stress test.
Data exclusions	no data were excluded
Reproducibility	While we did not repeat our study, the findings from the heat stress test are consistent with previous published work on the heat tolerance of these corals.
Randomization	Colonies were collected at least 10 m apart to avoid collecting clones.<br>One fragment from each of the 11 parent colonies was present in the six temperature treatments (n=11 per treatment), and fragments were randomly assigned to replicate tanks.<br>In order to assess the influence of long-term cold/warm acclimation and daily temperature variability on coral heat tolerance, a heat stress test was conducted at the end of the 9 months acclimation phase. Each of the treatments was split into a (i) control treatment maintained at ambient temperatures (genotypes 1-5, n=5) and (ii) heat stress treatment where temperatures were gradually increased to the known bleaching threshold of ~32°C (genotypes 6-11, n=6) (Fig. 1a, b). Parent colonies had randomly been assigned numbers prior to the start of the overall experiment, and at this stage, colonies #1-5 were assigned to the control treatment whereas colonies #6-11 were assigned to the heat stress treatment. A higher sample size was chosen for the stress treatment to account for potentially higher within-treatment variability. Due to logistical constraints associated with maintaining corals during these challenging long-term experiments, it was not possible to double the number of tanks to have full tank replication during the heat stress test; however, full tank replication was achieved for the critical 9-months acclimation phase.
Blinding	this is not applicable for coral mesocosm experiments

Did the study involve field work? ☒ Yes ☐ No

Field work, collection and transport

Field conditions	this was a laboratory study involving wild-collected animals (see below)
Location	We used the common, reef-building coral species Acropora aspera from the macrotidal Kimberley region in NW Australia as study organism (Fig. 1b). Intertidal populations of this species are known to have a superior heat tolerance promoted by the extreme environmental conditions in this region, and are dominated by generalist symbionts from the genus Cladocopium (previously clade C). Eleven visibly healthy colonies were collected in April 2016 from the intertidal at Shell Island, Cygnet Bay (16°28’45.8”S, 123°2’41.3”E). Colonies were collected at least 10 m apart to avoid collecting clones. This site features a tidal range of up to 8 m (Fig. 1b); thus, intertidal corals regularly experience prolonged aerial exposure, high light levels and extreme daily temperature fluctuations of up to 7°C, with short-term maxima of up to 37°C. Monthly average temperatures range from ~25-31°C (Fig. 1a, Supplementary Table 1), and the bleaching threshold was experimentally established to be ~32°C.
Access and import/export	Relevant permits were obtained from the Department of Fisheries (exemption no 2549, date of issue 3 March 2015).
Disturbance	Acropora corals are highly abundant, fast-growing corals and only a small amount of material was collected.

Reporting for specific materials, systems and methods

We require information from authors about some types of materials, experimental systems and methods used in many studies. Here, indicate whether each material, system or method listed is relevant to your study. If you are not sure if a list item applies to your research, read the appropriate section before selecting a response.

Materials & experimental systems

n/a	Involved in the study	
☒	☐	Antibodies
☒	☐	Eukaryotic cell lines
☒	☐	Palaeontology
☐	☒	Animals and other organisms
☒	☐	Human research participants
☒	☐	Clinical data

Methods

n/a	Involved in the study	
☒	☐	ChIP-seq
☒	☐	Flow cytometry
☒	☐	MRI-based neuroimaging

Animals and other organisms

Policy information about studies involving animals; ARRIVE guidelines recommended for reporting animal research

Laboratory animals	the study did not involve laboratory animals
Wild animals	We used the common, reef-building coral species Acropora aspera from the macrotidal Kimberley region in NW Australia as study organism. Intertidal populations of this species are known to have a superior heat tolerance promoted by the extreme environmental conditions in this region, and are dominated by generalist symbionts from the genus Cladocopium (previously clade C). Eleven visibly healthy colonies were collected in April 2016 from the intertidal at Shell Island, Cygnet Bay (16°28’45.8”S, 123°2’41.3”E).
Field-collected samples	Colonies were live-shipped to the University of Western Australia and maintained in indoor, flow-through aquaria at the Watermans Bay seawater facility at ~29°C to facilitate recovery and acclimation to tank conditions. Temperatures were kept constant within ~1°C. From mid-June until the end of July 2016, temperatures were adjusted twice a month to mimic seasonal temperatures at the collection site (Supplementary Table 1). Light was provided on a 12:12 hour light:dark cycle, following a natural diurnal light cycle with gradual increases up to 560 µmol m-2 s-1 at noon. Corals were fed twice a week with live brine shrimp. Further details on the mesocosm tank setup are given in the Supplementary Information. In July 2016, each colony was fragmented into 6 pieces of 5-10 cm which were glued onto pre-labelled plastic tiles. Coral fragments were allowed to recover for ~2 weeks prior to the start of the experiment at the beginning of August 2016.
Ethics oversight	ethics approval is not required for corals

Note that full information on the approval of the study protocol must also be provided in the manuscript.
